# *Mycoplasma genitalium* Infection in Adults Reporting Sexual Contact with Infected Partners, Australia, 2008–2016 

**DOI:** 10.3201/eid2311.170998

**Published:** 2017-11

**Authors:** Josephine B. Slifirski, Lenka A. Vodstrcil, Christopher K. Fairley, Jason J. Ong, Eric P.F. Chow, Marcus Y. Chen, Timothy R.H. Read, Catriona S. Bradshaw

**Affiliations:** Monash University, Melbourne, Victoria, Australia (J.B. Slifirski, L.A. Vodstrcil, C.K. Fairley, J.J. Ong, E.P.F. Chow, M.Y. Chen, T.R.H. Read, C.S. Bradshaw);; Melbourne Sexual Health Centre, Alfred Health, Melbourne (L.A. Vodstrcil, C.K. Fairley, J.J. Ong, E.P.F. Chow, M.Y. Chen, T.R.H. Read, C.S. Bradshaw)

**Keywords:** *Mycoplasma genitalium*, sexual contacts, partner management, contact tracing, men who have sex with men, MSM, Melbourne, Victoria, Australia, bacteria, sexually transmitted infections

## Abstract

Data on the likelihood of *Mycoplasma genitalium* infection in sexual contacts, particularly for men who have sex with men (MSM), are needed to form an evidence base for guidelines. We conducted a cross-sectional analysis of patients attending a sexual health clinic in Melbourne, Victoria, Australia, during 2008–2016. We calculated the proportion of contacts with *M. genitalium* infection and determined factors associated with infection. Among those patients reporting sexual contact with an *M. genitalium*–infected person, 48.2% of women, 31.0% of heterosexual men, and 41.7% of MSM were infected. Among heterosexual contacts, women were twice as likely to be infected; among MSM, rectal infection was more common than urethral infection; and among persons within heterosexual partnerships, concordance of infection was high. High positivity among female and MSM contacts and high concordance in heterosexual partnerships provide some justification for presumptive treatment; however, clinicians should consider antimicrobial drug resistance and toxicity of quinolones.

*Mycoplasma genitalium* is an established sexually transmitted pathogen that causes nongonococcal urethritis, and recent evidence indicates that it increases the risk for cervicitis, pelvic inflammatory disease, preterm delivery, and spontaneous abortion (*1*,*2*). The estimated prevalence of *M. genitalium* infection is 1%–3% in men and women, according to community-based studies from the United Kingdom, United States, Australia, and Scandinavia (*3*–*7*). Early diagnosis and effective treatment are therefore important in preventing sequelae and ongoing transmission, particularly the transmission of drug-resistant strains to sex partners.

Published data are limited regarding the likelihood of transmission of *M. genitalium* and the proportion of persons who are likely to be infected after contact with an infected sex partner. Several small studies, with the number of participants ranging from 8 to 88, have examined the proportion of persons infected when their partner has a confirmed *M. genitalium* infection, with results indicating a range of 20.6%–66.7% (*8*–*14*). However, the CIs are broad, and greater precision would provide a more accurate evidence base for partner-notification guidelines and clinical practice. To our knowledge, no published estimates of the likelihood of *M. genitalium* infection in contacts of infected men who have sex with men (MSM) are available. Studies of *M. genitalium* in MSM attending clinics report rectal infection prevalence of 1%–5% in predominantly asymptomatic men, whereas a recent study of MSM in Australia with proctitis found 8% of HIV-negative MSM and 20% of HIV-positive MSM had rectal *M. genitalium* infection (*15*–*19*).

Treatment guidelines are inconsistent about the need for presumptive treatment of sexual contacts of *M. genitalium*–infected patients; guidelines in the United States and United Kingdom do not recommend presumptive treatment, whereas guidelines in Australia do (*20*–*22*). Potential disadvantages of presumptive treatment include cost, unnecessary use of antimicrobial drugs, and risk for adverse effects, particularly from fluoroquinolones used for macrolide-resistant *M. genitalium*. The potential advantages are that early treatment might prevent reinfection of the index patient or transmission to others and prevent sequelae. The higher the likelihood of infection in a contact of a person with confirmed infection, the stronger the argument for presumptive treatment. Presumptive treatment for chlamydial infection, a sexually transmitted infection with similar characteristics to *M.* *genitalium* infection, is recommended based on prevalence estimates of 36%–68% among contacts of sex partners with confirmed chlamydial infection (*23*–*26*).

We performed a retrospective analysis of clinical records of patients attending a large urban sexual health service in Melbourne, Victoria, Australia, who reported sexual contact with a partner with diagnosed *M. genitalium* infection*.* We aimed to determine the proportion of cases with *M. genitalium* and the factors associated with infection in women, heterosexual men, and MSM.

## Methods

We conducted our study at the Melbourne Sexual Health Centre, the largest public STI clinic in Victoria, Australia. Starting August 2008, the clinic began treating sexual contacts of *M. genitalium*–infected patients and recording these cases in the clinic database. We defined a contact as someone who reported anal or vaginal sex with or without condoms with a person reporting a recent diagnosis of *M. genitalium* infection. Persons reporting only oral sex did not meet our definition of a contact. Persons were included at first report of being a contact, and repeat presentations were excluded. MSM were defined as men reporting any sex with men within the preceding 12 months.

We tested contacts by using an in-house real-time PCR assay targeting the 16s rRNA gene (*27*). Men were predominantly tested by using a first-pass urine sample, rarely with a urethral swab, and with an anorectal swab if anal sex was reported. Women were tested using a high vaginal swab or cervical swab, but a first-pass urine sample was used if patients preferred, and an anorectal swab was taken if anal sex was reported. We did not test for pharyngeal *M. genitalium* in men or women because of the absence of published evidence for infection at this anatomic site (*15*,*28*).

We recorded all sexual contacts of the *M. genitalium*–infected patients who attended the clinic during August 2008–July 2016 in the clinic database. We extracted demographic, behavioral, laboratory, and clinical data from the clinic’s electronic medical records, including number and sex of sex partners, sexual practices within the preceding 3 months, whether these partners were considered casual or regular partners, and consistency of condom use. Data were routinely obtained by clinicians and computer-assisted self-interview. Signs and symptoms among men reporting sexual contact with an infected person were urethral discharge, irritation, dysuria, rectal pain, and bleeding. Signs and symptoms among women reporting sexual contact with an infected person were abnormal vaginal discharge, dysuria, abnormal bleeding, and lower abdominal pain.

We performed statistical analyses by using Stata version 12 (StataCorp LLP, College Station, TX, USA). We calculated the proportion of contacts infected with *M. genitalium*, including 95% CIs, for 3 groups: women, heterosexual men, and MSM. We examined factors associated with infection for 2 groups: 1) heterosexual men and women, and 2) MSM. We conducted univariate logistic regression for each group by using demographic and behavioral characteristics as independent variables and detection of *M. genitalium* as the dependent variable. We treated age as a binary variable, with a cutoff at 27 years for all groups. We also treated the number of sex partners as a binary variable, with a cutoff at 1 for all groups. We used the χ^2^ or Fisher exact test, where appropriate, to assess the statistical significance of these associations. We calculated crude odds ratios (ORs) with 95% CIs, entered variables with p values <0.10 in the univariate analysis in the multivariate analysis by using forward stepwise logistic regression, and calculated adjusted ORs (aORs) with 95% CIs. In multivariate analyses, we omitted the binary variable for number of partners because of collinearity with the variable indicating whether the notifying partner was a regular or casual partner. Because some MSM had urine tests, others had rectal swabs, and some had both, we entered each test, rather than each person, into a multivariate model examining risk factors for infection in MSM by using robust SEs to account for clustering around persons.

In a subset of contacts, we were able to identify the referring partner in the clinic’s electronic medical record system. If this partner’s *M. genitalium* infection was diagnosed at the clinic within 40 days of the contact’s presentation, we included the contact in a further analysis of sexual partnerships (dyads).

## Results

During the study period, a total of 441 presentations to the clinic were made by patients reporting sexual contact with a person with *M. genitalium* infection (Figure). We excluded repeat presentations by the same person (n = 25), those missing laboratory test results (n = 16), those missing >50% of the queried behavioral data (n = 1), and those not meeting our definition of a contact (n = 22). These exclusions left 377 (85.5%) persons (139 women, 126 heterosexual men, and 112 MSM) for analysis.

**Figure:**
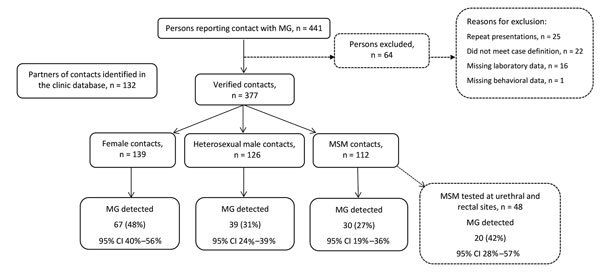
Flowchart for 441 persons examined at Melbourne Sexual Health Centre who reported sexual contact with a *Mycoplasma genitalium*–infected partner, Melbourne, Victoria, Australia, August 2008–July 2016. Dashed lines indicate persons excluded for analysis or subanalysis. MG, *Mycoplasma genitalium*; MSM, men who have sex with men.

### Baseline Characteristics of Study Population

We summarized baseline characteristics of the study population (Table 1). The median age of 139 female contacts was 26 years (interquartile range [IQR] 22–32 years). A total of 132 (95.0%) women were heterosexual, whereas 7 (5.0%) reported sex with men and women. The median age among 126 heterosexual male contacts of *M. genitalium*–infected patients was 28 years (IQR 24–35 years). The median age among 112 MSM contacts of *M. genitalium*–infected patients was 29 years (IQR 25–36 years). Most contacts in all 3 groups reported that their notifying partner was their regular partner, and most reported <100% condom use during the preceding 3 months.

**Table 1:** Baseline characteristics of 377 persons seen at Melbourne Sexual Health Centre who reported sexual contact with an *Mycoplasma genitalium*–infected partner, Melbourne, Victoria, Australia, August 2008–July 2016*

Characteristic	Women, n = 139	Heterosexual men, n = 126	MSM, n = 112
Age, y, median (IQR)	26 (22–32)	28 (24–35)	29 (25–36)
No. sex partners in preceding 3 mo†			
1	74 (53.6)	52 (41.9)	37 (35.6)
>2	64 (46.4)	72 (58.1)	67 (64.4)
Condom use with all sex partners in preceding 3 mo			
100%	11 (8.0)	6 (4.9)	15 (14.4)
<100%	126 (92.0)	117 (95.1)	89 (85.6)
Nature of relationship with the notifying partner			
Casual	28 (20.9)	36 (29.0)	38 (36.2)
Regular	106 (79.1)	88 (71.0)	67 (63.8)
Condom use with the notifying partner in preceding 3 mo			
100%	15 (11.6)	6 (4.9)	15 (14.9)
<100%	114 (88.4)	117 (95.1)	86 (85.1)

*Values are no. (%) unless otherwise specified. IQR, interquartile range; MSM, men who have sex with men.  †Number of sex partners does not include female sex partners for female contacts or MSM contacts.

### *M. genitalium* Infection in Sexual Contacts

#### Heterosexual Women and Men

Because *M. genitalium* positivity did not significantly differ between cervical or high vaginal swabs or first-pass urine samples among women (50.0% vs. 46.1%; p = 0.643), we combined these samples for our analysis. The overall proportion of female contacts in whom *M. genitalium* was detected was 48.2% (95% CI 39.7%–56.8%). The proportion of heterosexual male contacts in whom urethral *M. genitalium* was detected was 31.0% (95% CI 23.0%–39.8%), which was significantly lower than the proportion of female contacts infected (p = 0.004).

#### MSM

The proportion of MSM contacts in whom *M. genitalium* was detected overall was 26.8% (95% CI 18.9%–36.0%). However, only 48 (42.9%) MSM were tested at both anatomic sites; 48 (42.9%) were tested only at the urethra, and 16 (14.3%) were tested only at the rectum. Of the 48 MSM contacts tested only at the urethra, 3 had *M. genitalium* detected (6.3%, 95% CI 1.3%–17.2%). In contrast, of the 16 MSM contacts tested only at the rectum, 7 had *M. genitalium* detected (43.8%, 95% CI 19.8%–70.1%). Of the 48 MSM contacts tested at both anatomic sites, 20 had *M. genitalium* detected (41.7%, 95% CI 27.6%–56.8%), with most (17/20) of these infections being rectal infections. Overall, 8 of 96 urethral sites tested for *M. genitalium* were positive (8.3%, 95% CI 4.3%–15.6%), compared with 24 of 59 rectal sites (40.7%, 95% CI 29.1%–53.4%).

### Factors Associated with Having *M. genitalium* Infection

#### Heterosexual Female and Male Contacts

We examined potential predictors of *M. genitalium* infection among heterosexual women and men (Table 2). Factors associated with being infected with *M. genitalium* on univariate analysis included female sex (p = 0.004), having a regular partner as the notifying partner (p = 0.013), and having >2 sex partners in the preceding 3 months (p = 0.024). Factors that were significantly associated with being infected were included in a multivariate analysis; number of sex partners was not included because it was highly correlated with having a regular partner as the notifying partner, and condom use with the notifying partner was included given the protective effect of condoms against STI acquisition. Heterosexual contacts were more likely to be infected with *M. genitalium* if they were women (aOR 2.18, 95% CI 1.28–3.71) and the notifying partner was a regular sex partner (aOR 2.13, 95% CI 1.09–4.14). Contacts reporting <100% condom use with their notifying partner were 2.72 times more likely to have *M. genitalium* diagnosed*,* although this difference was not statistically significant (p = 0.066). The presence of any urethral discharge, irritation, or dysuria was associated with detection of *M. genitalium* in heterosexual men (OR 3.26, 95% CI 1.24–8.58). Symptoms were not associated with detection in women or in the combined (male and female) heterosexual model (Table 2).

**Table 2:** Potential predictors of *Mycoplasma genitalium* infection among heterosexual men and women seen at Melbourne Sexual Health Centre who reported sexual contact with an *M. genitalium*–infected partner, Melbourne, Victoria, Australia, August 2008–July 2016*

Characteristic	No.	Infected, no. (%)	Not infected, no. (%)	Unadjusted OR (95% CI)	p value	aOR† (95% CI)	p value
Total	265						
Age of sex partner, y‡							
>27	141	53 (37.6)	88 (62.4)	1.0			
<27	124	53 (42.7)	71 (57.3)	0.81 (0.49–1.32)	0.393	–	–
Sex							
M	126	39 (31.0)	87 (69.0)	1.0		1.0	
F	139	67 (48.2)	72 (51.8)	**2.08 (1.25–3.43)**	**0.004**	**2.18 (1.28–3.71)**	**0.004**
No. of sex partners in preceding 3 mo							
1	126	59 (46.8)	67 (53.2)	1.0			
>2	136	45 (33.1)	91 (66.9)	**0.56 (0.34–0.93)**	**0.024**	–	–
Nature of relationship with the notifying partner							
Casual	64	17 (26.6)	47 (73.4)	1.0		1.0	
Regular	194	86 (44.3)	108 (55.7)	**2.20 (1.18–4.10)**	**0.013**	**2.13 (1.09–4.14)**	**0.026**
Condom use with notifying partner in preceding 3 mo							
100%	21	5 (23.8)	16 (76.2)	1.0		1.0	
<100%	231	97 (42.0)	134 (58.0)	2.32 (0.13–1.27)	0.113	2.72 (0.93–7.91)	0.066
Symptoms in men§							
No	99	25 (25.3)	74 (74.7)	1.0			
Yes	21	11 (52.4)	10 (47.6)	**3.26 (1.24–8.58)**	**0.017**	–	–
Symptoms in women¶							
No	93	45 (48.4)	48 (51.6)	1.0		–	–
Yes	24	14 (58.3)	10 (41.7)	1.49 (0.60–3.70)	0.387		

*Bold text indicates a statistically significant association (p<0.05). Up to 7% of participants have missing data for some variables. aOR, adjusted odds ratio; MSM, men who have sex with men; OR, odds ratio. †Adjusted for sex, nature of relationship with notifying partner, and reported condom use (100% vs. <100%). ‡The median age across all female, heterosexual male, and MSM contacts was 27 years. §Discharge, dysuria, or urethral irritation; 6 men with urethral chlamydia excluded. In the combined heterosexual population symptoms were not significantly associated with infection and were not included in the multivariate model. ¶Vaginal discharge, dysuria, abnormal bleeding, or lower abdominal pain; 22 women with candidiasis, bacterial vaginosis, chlamydia, or urinary tract infections excluded.

#### MSM Contacts

Because most MSM were tested only at the urethra or the rectum, we based our analysis on the anatomic sites tested for *M. genitalium* rather than persons. By including each urethral (n = 96) and rectal (n = 59) test as individual observations within the dataset, we observed that 112 MSM contacts had 155 separate tests for *M. genitalium*. Factors that were significantly associated with infection on univariate analysis (or that were of borderline significance) included reporting having 1 sex partner in the preceding 3 months (p = 0.071), reporting <100% condom use with the notifying partner in the preceding 3 months (p = 0.061), and being tested at the rectal site (p<0.001) (Table 3). Including these 3 factors in a multivariate analysis, MSM contacts had an 8-fold increase in probability of *M. genitalium* infection if they were tested at the rectum instead of the urethra (aOR 8.39, 95% CI 3.14–22.42). In separate univariate analyses, restricted to persons tested at the relevant site, symptoms were not associated with detection of *M. genitalium* (Table 3).

**Table 3:** Factors associated with detection of *Mycoplasma genitalium* infection among MSM examined at Melbourne Sexual Health Centre who reported sexual contact with an *M. genitalium*–infected partner, Melbourne, Victoria, Australia, August 2008–July 2016*

Characteristic	No.	Infected, no. (%)	Not infected, no. (%)	Unadjusted OR (95% CI)	p value	aOR† (95% CI)	p value
Total tests	155						
Age of sex partner, y‡							
>27	51	11 (21.6)	40 (78.4)	1.0			
<27	104	21 (20.2)	83 (79.8)	0.92 (0.41–2.06)	0.840	–	–
No. of sex partners in preceding 3 mo							
1	51	15 (29.4)	36 (70.6)	1.0		1.0	
>2	95	16 (16.8)	79 (83.2)	0.49 (0.22–1.06)	0.071	0.62 (0.25–1.54)	0.303
Nature of relationship with the notifying partner							
Casual	55	11 (20.0)	44 (80.0)	1.0			
Regular	90	21 (23.3)	69 (76.7)	1.22 (0.57–2.62)	0.615	–	–
Condom use with the notifying partner in preceding 3 mo							
100%	20	1 (5.0)	19 (95.0)	1.0		1.0	
<100%	120	31 (25.8)	89 (74.2)	6.62 (0.92–47.85)	0.061	5.41 (0.70–41.82)	0.105
Anatomic site tested							
Urethra	96	8 (8.3)	88 (91.7)	1.0		1.0	
Rectum	59	24 (40.7)	35 (59.3)	**7.54 (3.08–18.45)**	**<0.001**	**8.39 (3.14–22.42)**	**<0.001**
Urethral symptoms§							
No	78	6 (7.7)	72 (92.3)	1.0			
Yes	12	1 (8.3)	11 (92.2)	1.09 (0.12–9.94)	0.938	–	–
Rectal bleeding or pain¶							
No	49	19 (38.8)	30 (61.2)	1.0			
Yes	2	1 (50.0)	1 (50.0)	1.58 (0.09–26.78)	0.752	–	–

*Bold text indicates a statistically significant association (p<0.05). Up to 7% of participants have missing data for some variables. aOR, adjusted odds ratio; MSM, men who have sex with men; OR, odds ratio. †Adjusted for number of partners in the previous 3 months, reported condom use (100% vs. <100%), and anatomic site tested.  ‡The median age across all female, heterosexual male, and MSM contacts was 27 years.  §Discharge, dysuria, or urethral irritation. Analysis restricted to urine samples only after excluding coinfection with *Chlamydia trachomatis* (n = 3). ¶Analysis restricted to rectal swabs only after excluding coinfection with *Chlamydia trachomatis* (n = 2) and *Neisseria gonorrhoeae* (n = 6).

### *M. genitalium* Infection in Sexual Partnerships (Dyads)

Of 377 contacts, 132 (35%) reported having been notified by a partner who could be identified in the clinic’s electronic medical record system. A total of 120 (91%) partnerships fulfilled the inclusion criteria for further analysis. In 86 heterosexual dyads, the median time between the contact and their partner being tested for *M. genitalium* was 8 days (IQR 6–16 days); in 34 MSM dyads, it was 7 days (IQR 4–11 days). Forty of 86 heterosexual dyads were concordant for *M. genitalium* infection (46.5%, 95% CI 36.4%–57.0%). Nine of 34 MSM dyads were concordant for infection (26.5%, 95% CI 14.6%–43.1%); however, few MSM dyads were tested for *M. genitalium* at both urethral and rectal sites. Of 34 MSM notifying partners that were identified, 29 (85.3%) had a history of urethral *M. genitalium* infection and 5 (14.7%) had a history of rectal *M. genitalium* infection.

## Discussion

In this study, a high proportion of persons reporting contact with an *M. genitalium*–infected partner were infected, including 48% of women, 31% of heterosexual men, and 42% of MSM tested at both the rectum and urethra. The sample size for this study exceeds the combined total of sample sizes in previously published studies, adding precision to estimates of the probability of infection and transmissibility of *M. genitalium* between sex partners (*8*–*14*). These findings will inform guidelines for the management of sexual contacts of *M. genitalium*–infected patients and provide an evidence base for informed discussion between clinicians and their patients regarding the appropriateness of presumptive treatment for contacts of infected patients or recommending testing and return for treatment.

In this study, among heterosexual contacts, women were twice as likely as men to be infected with *M. genitalium*, after adjusting for condom use and nature of relationship. This finding could be attributable to the female genital tract’s greater susceptibility to STIs, with the larger surface area of the cervico-vaginal mucosa compared with the urethral mucosa (*29*), and female sex hormones thought to enhance susceptibility to STIs (*30*). Heterosexual contacts notified by a regular partner were twice as likely to be infected, suggesting that multiple sexual acts or events of exposure might increase risk for acquisition of *M. genitalium.* Less than 100% condom use for penile-vaginal sex with a regular partner appeared to double the risk for *M. genitalium* infection among heterosexual contacts, and although this increased risk was not significant (p = 0.07), it does suggest that condoms provide protection against *M. genitalium* infection, as has been shown for other bacterial STIs. Concordance for *M. genitalium* infection in heterosexual dyads in which both partners were tested at our service was 47%, reflecting the high risk for concurrent infection in heterosexual partnerships. Overall, the prevalence of *M. genitalium* infection in heterosexual men and women was within the range reported for chlamydial infection in published studies (*23*–*26*).

The prevalence of *M. genitalium* that was observed among contacts in this study is substantially higher than the prevalence reported in comparable study populations in Melbourne. Reported prevalence estimates from these studies were 2.4% (95% CI 1.5%–3.3%) in young women attending clinics, including the site of this study (*4*); 1.3% (95% CI 0.3%–3.7%) in urine samples from asymptomatic heterosexual men (*31*); and (2.1%; 95% CI 1.1%–3.6%) in rectal swabs and urine samples from asymptomatic MSM (*15*), all of which are much lower than the respective prevalence estimates reported in our study of 48.2% (95% CI 39.7%–56.8%), 31.0% (95% CI 23.0%–39.8%), and 41.7% (95% CI 27.6%–56.8%).

MSM contacts had a similar likelihood of being infected with *M. genitalium* as women when they were tested at both the urethra and the rectum. This study highlights the importance of rectal testing for *M. genitalium* in MSM. Urethral positivity was only 8% in MSM, compared with 31% in heterosexual men. However, overall rectal positivity was high at 38%, and when MSM were tested at both urethral and rectal sites, 42% were positive for *M. genitalium*, and most of these had rectal infections. The clinic records do not indicate why some men were not tested at both sites. The higher rate of rectal infection compared with urethral infection is consistent with studies of chlamydial infection among MSM but is also likely to be influenced by the notifying partner’s reason for seeking care. When this factor was examined among MSM dyads, 29 of 34 MSM notifying partners sought care for urethral infections, suggesting that urethral infections might be more likely than rectal infections to cause symptoms.

Our study has several limitations. The study is retrospective and relies on self-report of exposure to infection without laboratory confirmation. As such, the data reflect the prevalence of infection only among those persons who seek care reporting exposure to *M. genitalium* rather than among all of those exposed. We have no information on contacts of infected patients who did not attend the clinic, and these persons are likely to be systematically different from those who did seek out testing and treatment. These findings might also not be generalizable to non–STI clinic populations or to other populations with a different background prevalence of *M. genitalium* infection. Although we considered the notifying partner the index patient for analytical purposes, we cannot ascertain the transmission direction between sex partners or whether transmission occurred through a third person. Sexual behavioral data were self-reported and hence subject to recall bias. The most notable limitation was the lack of dual-site testing for MSM contacts, which limited our ability to report precise estimates of infection among MSM and to examine concordance in MSM dyads.

Presumptive treatment of sexual contacts reduces the risk for reinfection and is recommended for STI syndromes such as nongonococcal urethritis (*20*). In the contacts of *M. genitalium­*–infected persons in this study, presumptive treatment would have treated 1 infection for every 2–3 treatments. However, the decision to recommend presumptive treatment must also take into account potential harms and benefits to the contact and their sex partners. Although heterosexual men had a slightly lower prevalence of positivity, presumptive treatment might be more important in reducing the risk for serious sequelae, such as pelvic inflammatory disease, in female partners. 

The alternate approach of treating contacts only after confirmation of *M. genitalium* infection represents better stewardship of antimicrobial drugs but relies on access to sensitive testing practices and a high rate of return of patients to be effective. An important consideration before presumptively treating contacts for *M. genitalium* infection is the increasing prevalence of macrolide resistance, which is >40% in Europe, Japan, and the United States and >75% among MSM in Australia (*32*–*35*). Furthermore, macrolide resistance is selected in 12%–18% of seemingly susceptible infections after treatment with 1 g azithromycin and extended azithromycin regimens (*35*). Presumptive use of macrolides for *M. genitalium*–infected contacts might therefore not only be ineffective in those patients with detectable resistance but also contribute to development and spread of resistance, particularly in asymptomatic contacts who believe they have been effectively treated. The only recommended treatments for macrolide-resistant *M. genitalium* are fourth-generation fluoroquinolones, which are expensive and can cause tendinopathy, neuropathy, and adverse central nervous system effects, which are major considerations for determining their use in persons who do not have confirmed infection. Overall, a prudent approach entails managing sexual contacts according to the informed preferences of the person and, if known, the resistance status of the notifying partner. The results of our study provide an evidence base for informed discussions between clinicians and patients at risk for infection and can inform international treatment and partner-notification guidelines.
